# Gut Microbiota in IBD: The Beneficial and Adverse Effects of Diet and Medication

**DOI:** 10.3390/nu18010009

**Published:** 2025-12-19

**Authors:** Aidan Eric Juhl, Morten Westfall, Betina Hebbelstrup Jensen, Hengameh Chloé Mirsepasi-Lauridsen

**Affiliations:** 1 Department of Bioengineering, Florida Gulf Coast University, Fort Myers, FL 33965, USA; 2 Department of Physics, Chemistry, and Pharmacy, University of Southern Denmark, 5000 Odense, Denmark; 3 Center for General Practice, Department of Public Health, University of Copenhagen, 1353 Copenhagen, Denmark; betina.hebbelstrup@sund.ku.dk; 4 Department of Gastroenterology, Copenhagen University Hospital Hvidovre, 2650 Hvidovre, Denmark; 5 Department of Infectious Diseases, Copenhagen University Hospital Hvidovre, 2650 Hvidovre, Denmark; 6 Department of Research and Development, 1Health Gut Inn Balance, 2680 Solroed Strand, Denmark

**Keywords:** inflammatory bowel disease, diet, medication, ulcerative colitis, Crohn’s disease

## Abstract

Background: Inflammatory bowel disease (IBD) is a global disease with a considerable increase in prevalence and the impact on the health and well-being of patients suffering from this condition is vast. Diet has been suspected of being a contributor to IBD severity as well as intake of antibiotics. Methods: A literary search was conducted on the most recent studies on the subject of IBD, diet, and medical treatment to identify high-quality research findings within this area of research. Research published within the last decade was prioritized. Studies in English language were included in the search, and the knowledge gained was synthesized in the review. Results: Dietary patterns, specifically intake of Westernized diets, were associated with increased inflammation and increased disease severity in patients suffering from IBD, specifically patients suffering from Crohn’s disease (CD). A co-administration of pre- and probiotics was found to contribute to disease remission in ulcerative colitis patients, however, to a lesser extent in patients with CD. A bidirectional effect on the intestinal microbiome was seen as a result of intake of the medicines used for the treatment of IBD patients, which affects both bioavailability of the drug and efficacy of the treatment. The baseline composition of the intestinal microbiome in IBD patients dictates their response to the different treatments. Conclusions: Diet and medical treatment both have a large impact on the architecture of the intestinal Microbiome in IBD patients and are, as such, both essential to understand to enable individualized and optimized treatment.

## 1. Introduction

Inflammatory bowel disease (IBD) is a chronic inflammatory disease of the gastrointestinal tract, which has traditionally been divided into Crohn’s disease (CD) and ulcerative colitis (UC) [1]. UC is a relapsing, chronic inflammatory disease that is restricted to the colon affecting only the mucosal membrane and is characterized by intermittent bloody diarrhea with pus, abdominal pain, weight loss, fatigue, and an urgent need of defecation. CD is a chronic, segmental, localized granulomatous disease that can affect the entire gastrointestinal tract, from the mouth to the anus, and is transmural. The symptoms are abdominal pain, chronic diarrhea—sometimes with blood—weight loss, ulcers in the mouth, and malabsorption. The etiology of IBD remains unknown; however, accumulating evidence suggests that a combination of dietary factors [2,3], intestinal dysbiosis [1,4], and abnormal immune response [5] may act as key triggers in disease development.

The annual incidence of IBD rates varies geographically, with 10.5 to 46.14 per 100,000 inhabitants in Europe, 1.37 to 1.5 per 100,000 inhabitants in Asia, 0.21 to 3.67 per 100,000 inhabitants in South America, and 7.3 to 30.2 per 100,000 inhabitants in North America [6]. The rising incidence of IBD among children and adolescents highlights the concern that oscillations in hormones may play a role as potential disease triggers [6]. A significantly higher regional prevalence of IBD has been observed in the Faroe Islands, with 106 cases per 100,000 inhabitants [7]. This raises the question of whether the differences in the IBD prevalence across regions are driven by diet, lifestyle, or genetic predisposition.

Human gut microbiota plays a central role in host metabolism, immunity, and neuroendocrine function [7]. The gut microbiome also influences the human hormone axis by modulating secretion of serotonin, insulin/leptin [8], cortisol, estrogen/testosterone [9], etc., which are speculated as a trigger of IBD among children and adolescents. It is well established that vaginally delivered newborns acquire their initial gut microbiota from their mother during birth and later through breastfeeding—both crucial for healthy gut development [10]. In contrast, infants delivered by cesarean section primarily acquire their early microbiota from the skin and surrounding hospital environment^10^ and are found to be more exposed to chronic diseases such as IBD and asthma [11].

Environmental factors—particularly diet [3] and medication [10]—play a major role in shaping the gut microbiota throughout life.

Although genetic predisposition is a well-established contributor to IBD pathogenesis—highlighted by variants in genes such as *NOD2*, *ATG16L1* [12], and *IL23R*, which influence innate immunity, autophagy, and host–microbiome interactions [12], the present manuscript is intentionally focused on dietary factors and pharmacological therapies as modulators of the gut microbiome. Genetic factors shape disease susceptibility and can modify microbial composition or therapeutic response; however, an in-depth analysis of gene–microbiome interactions are beyond the scope of this review.

Diets rich in plant-based fibers promote the growth of beneficial bacterial taxa such as *Faecalibacterium prausnitzii*, *Roseburia* spp., *Eubacterium rectale*, and *Bifidobacterium* spp., which produce short-chain fatty acids (SCFAs) including butyrate, acetate, and propionate. These metabolites are essential for maintaining intestinal barrier integrity, regulating immune homeostasis, and supporting overall gut health [9]. In contrast, high-fat, high-sugar, and low-fiber Western diets reduce microbial diversity and favor pro-inflammatory bacterial species, contributing to dysbiosis and metabolic inflammation in the gut [13].

Additionally, medication such as antibiotics [10], proton-pump inhibitors [10], and corticosteroids [11] affects the host microbiome. This review explores the dual impact of diet and medication on gut microbiota in IBD, emphasizing the mechanisms underlying the beneficial and detrimental effects.

## 2. Materials and Methods

A comprehensive review of the international scientific literature was conducted to assess the role of dietary patterns and commonly used medications in shaping gut microbiota in patients with IBD. The research focused on identifying the most recent evidence describing how diet and pharmacological treatments act as modulators of the gut microbiome across different stages of the disease, including both the pre-diagnostic and post-diagnostic phases.

### 2.1. Literature Search

The literature review primarily drew on PubMed, Scopus, and ScienceDirect. Search strategies were based on the combinations of the following keywords, informed by previous work in the field: “gut microbiota,” “gut dysbiosis,” “inflammatory bowel disease,” “medication and gut microbiota in IBD,” “role of diet in gut microbiota in IBD,” “nutritional management of IBD,” and “fecal microbiota transplantation in IBD”. This approach ensured comprehensive coverage of studies addressing both dietary and pharmacological modulation of the gut microbiome in IBD.

### 2.2. Search Strategy and Eligibility Criteria

The search strategy for this review was guided by the PICO framework (Population, Intervention, Comparator, Outcomes). Studies were included only if participants were followed post-treatment with medication or nutritional interventions, or during the course of a nutritional intervention.

Population: Human participants of any age are diagnosed with IBD.

Intervention: All variations in diets and medications as modulators of the gut microbiome in IBD were included. Fecal microbiota transplantation (FMT) was defined as any procedure transferring a fecal microbiota suspension from a healthy donor to a recipient for therapeutic purposes.

Comparator: Studies with a control group were preferred, though studies without controls were also considered when relevant.

Outcomes: Primary outcomes focused on gut microbiota modulation, while secondary outcomes included related clinical or biochemical measures.

Time frame: Priority was given to research published in the past 10 years to capture the most current findings; however, 32 of 128 references are older than 10 years and were included due to their fundamental contributions to IBD research.

Language: Only English-language articles were considered.

Study types: Original research, clinical studies, and reviews were included. Foundational knowledge was supplemented with textbooks and peer-reviewed scientific reviews.

A total of 145 articles met these selection criteria. Results from the selected studies were synthesized into a translational discussion addressing clinical implications and future perspectives for human IBD management.

### 2.3. Critical Evaluation of Included Studies

The methodological quality of included studies was evaluated using standard criteria: the Cochrane Risk of Bias for RCTs and the Newcastle–Ottawa Scale for observational studies. Most RCTs were rated as low to moderate risk of bias, while observational studies showed moderate risk due to small sample sizes and potential confounding factors. Limitations such as inconsistent outcome measures, lack of blinding, and short follow-up were noted. Overall, the evidence is informative and was interpreted with caution.

Most human studies on diet and medication in IBD have robust sample sizes (>100 patients).

FMT studies have smaller patient cohorts due to the novelty of the therapy; more clinical evidence is needed.

Three relevant animal model studies are included and discussed to provide mechanistic insights.

Potential biases, variability in interventions, outcome measurements, and differences between human and animal studies are addressed.

The implications of these methodological factors for interpreting results and overall evidence strength are discussed.

## 3. Diet as a Modulator of Gut Microbiota in IBD

### 3.1. Beneficial Dietary Patterns

The high prevalence of IBD in Western countries and newly industrialized countries suggests that environmental exposure, particularly diet, might contribute to the risk of IBD. It is well known that diet shapes the composition of the human gut microbiota and that host cells use microbial metabolites as energy sources and immunomodulatory agents to maintain the intestinal homeostasis. The symbiotic relationship within the gut microbiota is essential for human health [14]. Disruption of this balance, for instance, through increased consumption of Westernized diets, which are rich in fat and poor in fiber, can result in microbial dysbiosis with subsequent gut inflammation [15].

IBD profoundly affects nutrient metabolism and nutritional requirements, often leading to altered body composition such as low body mass index (BMI), reduced lean body mass [16], malnutrition [17], or in some cases, obesity and hypermetabolism [18]. Many IBD patients report that certain foods exacerbate their symptoms, although the triggers are highly individual [19]. Restricting fermentable carbohydrates could help alleviate non-inflammatory gastrointestinal symptoms for some patients. One of the largest long-term studies on diet and IBD—a 26-year prospective cohort involving 170,776 women—was conducted by researchers Ananthakrishnan et al. (2013) [20]. In this study, participants in the ’Nurses’ Health Study’ completed detailed dietary questionnaires every four years. The results showed that long-term dietary fiber intake, particularly fiber derived from fruit, was associated with a significantly lower risk of developing CD, but not UC. Another study by Lopes et al. (2023) investigated the relationship between lifestyle factors—including body mass index (BMI), smoking, physical activity, and the intake of fruits, vegetables, and dietary fiber—and the risk of developing IBD in adults [21]. Participants were drawn from three large U.S. prospective cohorts: the Nurses’ Health Study (year-1976, n = 72,290), the Nurses’ Health Study II (year-1989, n = 93,909) [22], and the Health Professionals Follow-up Study (n = 41,871). The findings demonstrated that adherence to a healthy lifestyle (based on healthy diet, increased physical activity, no smoking) was associated with approximately a 50% reduction in the incidence of IBD. These results were largely confirmed in three independent European cohorts, suggesting that a substantial proportion of IBD cases could potentially be prevented through lifestyle modifications [21].

#### Mediterranean Diet

**Figure 1:**
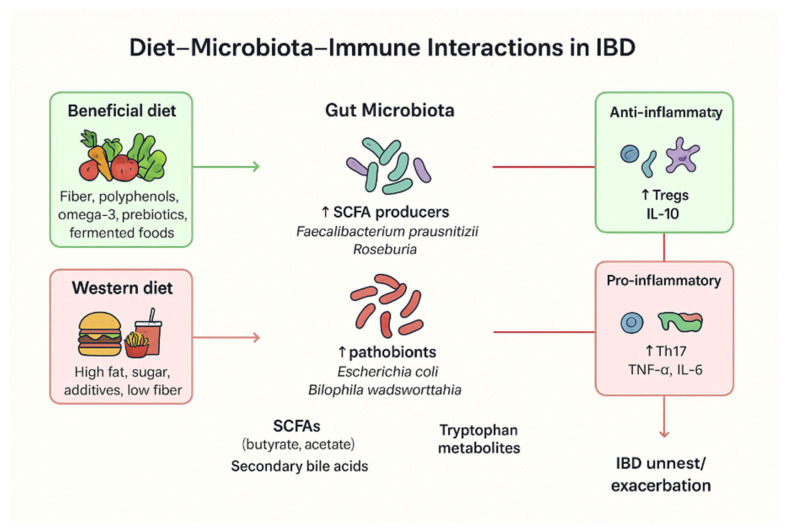
Overview of diet–microbiota–immune interactions in IBD. A beneficial diet—often associated with the Mediterranean diet and rich in omega-3 fatty acids, prebiotics, and other anti-inflammatory components—can increase the abundance of SCFA-producing bacteria, which in turn regulate anti-inflammatory markers such as Interleukin-10 (IL-10). In contrast, a Western diet high in fat and sugar is linked to an increased abundance of pathobionts such as *Escherichia coli*, which elevate pro-inflammatory factors including Tumor Necrosis Factor-alpha (TNF-α) and IL-6, thereby promoting IBD relapses.

The Mediterranean diet is based on a traditional diet from Mediterranean countries such as Spain, Italy, France, and Greece, which focuses on fresh non-boiled or non-fried food, high in unprocessed plant-based foods such as fruits, vegetables, nuts, wholegrain cereals, olive oil (saturated fat), moderate fish, and shellfish consumption. Furthermore, it contains low to moderate dairy such as cheese and yogurt, limited red meat, and processed foods [23] (see Figure 1).

Khalili et al., (2020) [24] conducted a 17-year prospective study involving 164 patients with CD and 395 patients with UC. The findings indicate that adherence to a Mediterranean diet was particularly beneficial for individuals with CD, as was associated with a significantly reduced risk of later CD onset [24].

Haskey et al. 2023 [25] conducted a study with 15 UC patients who were randomized to a Mediterranean diet, and 13 UC patients who were assigned to a Canadian habitual diet for 12 weeks. The study showed how the group randomized to the Mediterranean diet had reduced levels of fecal calprotectin and higher levels of short-chain fatty acids (SCFA) in stool samples [26]. In addition, the results showed that UC patients randomized to the Mediterranean diet had alterations in beneficial microbiota species such as *Alistipes finegoldi*, *Flavonifractor plauti*, and SCFA-producing *Ruminococcus bromii*, which are usually associated with healthy intestinal microbiomes.

Dietary items associated with anti-inflammatory effects include carrots, sweet potatoes, spinach, iceberg lettuce, romaine lettuce, potato chips, corn chips, popcorn, crackers, apples, oranges, grapes, prune juice, beer, wine, tea, and coffee. However, the anti-inflammatory impact of these foods on the intestinal microbiome appears to be highly individual among IBD patients [27]. A study of 194 UC patients in remission indicated that a diet consisting of low intake of meat products and reduced consumption of alcoholic beverages is linked to disease remission in UC patients [28].

### 3.2. Prebiotics and Probiotics

The efficacy of probiotics in treating patients suffering from IBD depends on the viability, stability, and ability of the bacteria to survive the acidic gastric environment in the ventricle and reach the intestine for colonization [29]. Combining treatment of IBD patients with both probiotics and prebiotics—such as psyllium, inulin, resistant starches, and dietary fibers—enhances the bacteria’s ability of survival and activity, promoting short-chain fatty acid (SCFA) production in the gut and will result in lowering of the luminal pH to support beneficial bacterial growth [30,31].

In UC patients, alterations in the gut environment, including elevated small intestinal pH (≈7.5 vs. 6.0 in healthy individuals) and variable colonic pH (2.3–5.5 vs. 6.7), contribute to a dysbiosis state [32,33]. This shift favors increased *Escherichia coli* expansion and reduces the levels of *Clostridiales*—which are both important SCFA producers—which will contribute to an increased disturbance of the microbial balance [31,33].

Clinical and experimental studies have evaluated the efficacy of several probiotics for treatment of UC patients, notably *E. coli* Nissle 1917 and VSL#3 (a mixture of the *Lactobacillus*, *Bifidobacterium*, and *Streptococcus* strains) [29]. In UC patients, treatment with *E. coli* Nissle 1917 showed immunomodulatory effects, including reduced mucosal T-cell infiltration and pro-inflammatory cytokines, while enhancing IL-10 secretion in the gut [4,34,35,36]. However, as this bacterium is a B2-phylogroup strain, it harbors *colibactin* genes linked to genotoxicity in the gut and possesses a colorectal cancer risk [37,38].

Randomized controlled trials of UC patients suggest that *E. coli* Nissle 1917 may be as effective as the conventionally used drug in UC patients mesalazine in maintaining UC disease remission [39,40,41]. Although the results in this study were inconsistent, larger studies are needed to confirm these findings [38]. VSL#3 has shown efficacy in sustaining disease remission in UC patients for more than six months [42,43], but long-term outcomes in the long-term health of UC patients remain uncertain.

Lactic acid bacteria (*Lactobacillus* spp.) produce bacteriocins with antimicrobial and immunoregulatory effects [44,45,46], while *Bifidobacterium*—a dominant genus in breast-fed infants—supports immune maturation and inhibits the invasion of enteric pathogens via organic acids and antimicrobial peptides [47,48]. Overall, microbiota-targeted nutritional strategies combining pre- and probiotics show considerable promise as adjunctive therapies in IBD patients, but further controlled validation is required (Table 1).

Evidence on the role of probiotics in treating patients suffering from CD remains limited. Some studies suggest that treatment with probiotic supplementation, particularly with *Bifidobacterium* species or multi-strain formulations, may help maintain disease remission when administered to the patients for three to six months [49].

The evidence for probiotics in IBD remains mixed, reflecting substantial heterogeneity in strain selection, dosing, and clinical endpoints. Strain-specific effects are common: combinations such as *VSL#3* have shown benefit in ulcerative colitis and pouchitis [50], whereas probiotics effects in CD often yield inconsistent results [50]. Dose–response relationships are poorly defined, and many studies use widely differing colony-forming unit (CFU) doses, making cross-study comparison challenging [50]. Although probiotics are generally considered safe, adverse events, including bloating, bacteremia in severely immunocompromised individuals, and rare systemic infections, have been reported [50]. More importantly, several well-controlled trials have reported no significant benefit compared with placebo, emphasizing that probiotics cannot yet be considered a reliable therapy for IBD management [50]. The variability in clinical outcomes underscores the need for standardized strain characterization, targeted indications, and mechanistic studies that can guide personalized microbial interventions.

### 3.3. Fecal Microbiota Transplantation (FMT)

FMT is an ancient therapy where beneficial microorganisms and substances from healthy donor stool are transferred to patients to restore balanced gut microbiota [51]. It contains not only bacteria but also viruses, metabolites like short-chain fatty acids (SCFAs), antimicrobial compounds, and other beneficial products that help repair the diseased intestinal environment.

The main challenges are ensuring safe donor screening [51,52,53,54,55], preserving anaerobic bacteria, and maintaining all beneficial components during production. Many current manufacturing methods such as exposure to oxygen [56], excessive dilution [57], centrifugation [57], or freeze-drying [58] can damage key microbes and reduce treatment effectiveness. Additives like cryoprotectants may also pose risks to the host [57].

FMT can be delivered by capsules, enema, endoscopy, or nasojejunal tube, depending on the disease location. Capsules are the most patient-friendly option for long-term treatment, especially in chronic conditions like UC and CD. However, high FMT capsule cost, inconsistent manufacturing methods, and safety concerns limit widespread use.

Although FMT has shown promising results in subsets of patients with IBD, safety concerns remain central to its clinical evaluation. Documented risks include pathogen transmission, particularly from multi-drug-resistant organisms (MDROs), which has led to safety alerts and stricter donor-screening requirements [53]. Rigorous donor selection protocols now involve extensive medical history review, serology, stool pathogen testing (including MDRO screening), and repeated assessments [55]. Long-term consequences of altering the gut microbiome remain uncertain, with concerns regarding metabolic, immunological, and oncological outcomes that may emerge years after treatment. Regulatory frameworks differ internationally: in the EU, FMT is regulated variably at the national level (ranging from tissue-based regulation to investigational medicinal product status), whereas the U.S. FDA currently classifies FMT as an investigational product except for recurrent *C. difficile* infection under enforcement discretion [50]. These issues highlight the need for standardized protocols, harmonized regulatory oversight, and high-quality randomized trials before widespread adoption of FMT in IBD. Under the upcoming EU Regulation on Substances of Human Origin (SoHO; Regulation (EU) 2024/1938) [59], which enters into force on 7 August 2027, stool banks and FMT treatment facilities will be classified as SoHO entities and regulated similarly to blood and tissue establishments.

The challenge with FMT is finding healthy stool donors and regularly screening for pathogenic microorganisms to avoid the risk of infection. Therefore, it is very important to follow the national or international guidelines regarding donor banking and donor screening [52,53,54,55].

Clinical studies show FMT can induce remission in UC patients, but results vary [60,61,62,63]. Long-term, repeated treatment with correct administration—using colonoscopy or oral capsule—seems more effective than single administrations using enema. It is also essential to consider the manufacturing method [64], as it can have a significant impact on clinical outcomes. The efficacy of FMT as any other medical treatment depends strongly on proper production processes, accurate dosing, and an appropriate duration of administration, which should be personalized according to the patient’s disease type and disease severity ((ICH Q7 (International Council for Harmonization), Good Manufacturing Practice for Active Pharmaceutical Ingredients), (WHO Essential Medicines (2019) Administration Frequency) [65,66].

High-quality clinical trials—especially using FMT capsules—are needed. Safe, standardized production in hospitals under strict guidelines is essential to make FMT reliable, accessible, and effective.

### 3.4. Harmful Dietary Patterns

The Westernized diet is linked to high-income, industrialized nations like the US and Western Europe, and it is rapidly spreading to developing and middle-income countries. A Westernized diet is based highly on ultra-processed beverages (UPBs), processed and ultra-processed foods (UPFs), red and processed meats, saturated fats, refined sugars, and high-calorie-density foods, while being low in dietary fiber, whole grains, fruits and vegetables, and other plant-based foods [67].

The consumption of ultra-processed foods (UPFs) including ready-to-eat soups, meals, sweets, breakfast cereals, baked goods, processed meats, ice creams, frozen desserts, and ultra-processed beverages (UPBs) such as flavored waters, concentrates, sports and energy drinks, coffee beverages, fruit juices, and carbonated soft drinks have all been associated with an increased risk of various non-communicable diseases, including IBD [68].

Despite these health risks, global consumption of UPFs and UPBs continues to rise, driven by major shifts in food production, processing, manufacturing, marketing, retail, and consumption practices, as well as the growing economic and political influence of large food corporations (“Big Food”) [68]. This trend is particularly pronounced in high-income countries, where increasing sales of UPF products parallel the rising prevalence of non-communicable diseases such as IBD. Increased consumption of UPBs and UPFs, which are high in salt, sugar, and fat, was observed to be associated with the development of CD cases in a meta-analysis comprising 1,068,425 participants each divided into five cohorts, where scientific findings were published between 2020 and 2022 [69] (see Figure 1).

The Westernized diet is strongly associated with the development of autoimmune and inflammatory diseases through multiple mechanisms:

(A) High fat intake contributes to obesity and modulates T-cell responses, promoting immune dysregulation [70].

(B) Excess sodium consumption (particularly from processed foods) induces pro-inflammatory immune phenotypes via osmotic stress pathways, including enhanced Th17 cell responses [71].

(C) Diets rich in fat and refined sugars alter the composition and structure of the gut microbiota, shifting it from a state of symbiosis to dysbiosis. This disruption triggers host immune activation and increase intestinal epithelial permeability [67]. The commensal intestine microbiota is crucial for elimination of pathogens in the gut. Alteration of the intestinal microbiota/dysbiosis leads to increased Bacteroidetes levels and a decreased beneficial bacteria/Firmicutes ratio [72].

Dietary items that have been associated with promoting inflammatory responses include various processed and red meats (such as hotdogs, bacon, hamburgers, beef, pork, lamb dishes, liver, canned tuna, shrimp, breaded fish, and other seafoods), as well as certain vegetables and plant foods (including corn, mixed vegetables, eggplant, celery, mushrooms, peppers, cucumbers, and fresh tomatoes, tomato juice, and tomato sauce). In addition, sugar-sweetened and artificially sweetened beverages such as cola, caffeine-free cola, Pepsi, other carbonated drinks containing caffeine and sugar, and low-calorie versions of these beverages have also been linked to increased inflammatory activity [27]. However, the impact of disease manifestations associated with the intake of above-mentioned inflammatory diets are highly individual among IBD patients.

Modulation of the gut microbiota in IBD alters the bioavailability of key vitamins and minerals, including vitamin K [73]. Reduced abundance of butyrate-producing *Clostridiales* species in active IBD leads to lower levels of fecal SCFA, diminishing the epithelial energy supply and the anti-inflammatory signaling while permitting the overgrowth of *E. coli* [74,75,76,77]. Micronutrient deficiencies (particularly iron, zinc, folate, and vitamin B12) contribute to anemia, which is one of the most common complications associated with IBD [78]. Zinc supports DNA synthesis and immune regulation [79], while iron, folate, and vitamin B12 are essential for erythropoiesisVitamin D deficiency is common in IBD, affecting gut barrier integrity via E-cadherin induction and promoting antimicrobial peptide production [80,81,82]. Nutritional therapy has been shown to result in superior mucosal healing compared with treatment with corticosteroids [83], and it may exert prebiotic effects by modulating the gut microbiota and restoring metabolic and immune balance, Table 1.

While inulin is well known and used as a prebiotic fiber that promotes *Bifidobacteria* growth and SCFA production, excessive intake of this can result in dysbiosis or inflammation in the gut, which was associated with hepatic inflammation and cholestasis in animal models with a high intake [84]. Dietary emulsifiers and food additives (e.g., polysorbate-80, carboxymethylcellulose) can disrupt the intestinal mucus layer in the gut, which can alter gut microbiota composition, and promote colitis-like inflammation in experimental animal models, Table 1 [85].

**Table 1:** Summary of dietary components and microbiota effects in inflammatory bowel disease (IBD).

Dietary Component	Microbiota Effects	Mechanistic/Metabolic Pathways	Impact on IBD Pathogenesis	Key References
Dietary fiber (prebiotics)	↑ Faecalibacterium prausnitzii, Roseburia, Bifidobacterium; ↑ microbial diversity	Fermentation → ↑ short-chain fatty acids (SCFAs), esp. butyrate; enhances epithelial integrity	Anti-inflammatory; promotes mucosal healing; ↓ flare frequency	Makki et al., 2018 [86]; Parada Venegas et al., 2019 [87]
High-fat (Western) diet	↓ Bacteroidetes; ↑ Firmicutes, Proteobacteria, Bilophila wadsworthia	↑ bile acids and endotoxins → triggers Th1/Th17 activation	Promote dysbiosis, intestinal permeability, chronic inflammation	Agus et al., 2018 [88]; Devkota et al., 2013 [89]
High-sugar diet	↓ Bacteroidetes, Akkermansia muciniphila; ↑ Enterobacteriaceae	Alters mucus layer, ↑ oxidative stress	Exacerbates colitis; increases gut permeability	Khan et al., 2020 [90]; Martinez-Medina et al., 2014 [91]
Animal protein (red/processed meat)	↓ Bacteroides, ↓ Lactobacillus	↑ Sulfide, ammonia, and nitroso compounds → epithelial damage	Correlated with increased relapse risk in UC	Jantchou et al., 2010 [92]; David et al., 2014 [93]
Polyunsaturated fatty acids (PUFAs)	Modulates Bacteroidetes/Firmicutes ratio	ω-3: anti-inflammatory; ω-6: pro-inflammatory eicosanoid production	ω-3 reduces inflammation; ω-6 aggravates it	Calder, 2015 [94]; Marion-Letellier et al., 2016 [95]
Polyphenols (plant-based foods)	↑ Lactobacillus, Bifidobacterium; ↓ Clostridium perfringens	Antioxidant, prebiotic effects; enhances SCFA production	Decreases inflammation, protects mucosa	Cardona et al., 2013 [96]; Etxeberria et al., 2013 [97]
Artificial sweeteners (sucralose, saccharin)	↓ SCFA producers; ↑ Bacteroides, Clostridiales	Alters microbial signaling and insulin response	Associated with dysbiosis, potential flare risk	Suez et al., 2014 [98]; Ruiz-Ojeda et al., 2019 [99]
Probiotic foods (yogurt, kefir, fermented vegetables)	↑ Lactobacillus, Bifidobacterium; competitive inhibition of pathobionts	Competitive inhibition, modulation of immune signaling	Shown to maintain remission and reduce symptoms	Ianiro et al., 2018 [100]; Derwa et al., 2017 [101]

↑ indicates an increase; ↓ indicates a decrease. Italicized text indicates bacterial taxa.

Dietary composition strongly influences gut microbial diversity and function. Fiber-rich and plant-based diets enhance the abundance of short-chain fatty acid (SCFA)-producing bacteria, promoting anti-inflammatory immune pathways and epithelial integrity. In contrast, Western-style diets high in fat and sugar reduce beneficial taxa and increase pro-inflammatory metabolites, contributing to dysbiosis and mucosal inflammation.

## 4. Medication and Gut Microbiota Interactions in IBD

Since nearly one in four adults worldwide use proton pump inhibitors (PPIs), PPI exposure represents an important confounder or effect modifier when examining the gut microbiota in patients with IBD [102]. Prolonged use of PPIs has been associated with reduced intestinal microbial diversity and reduced levels of beneficial anaerobes, while favoring enrichment of oral and upper gastrointestinal taxa such as *Streptococcus*, *Enterococcus*, and *Enterobacteriaceae* due to a less acidic environment in the upper GI tract. PPI-induced intestinal dysbiosis, which is associated with elevated gastric pH, may predispose to small intestinal bacterial overgrowth (SIBO) and altered metabolic activity [103,104], which is linked to dysbiosis.

The usage of non-steroidal anti-inflammatory drugs (NSAIDs) is very common but varies widely depending on age group, region, and indication. The studies show that NSAIDs promote a shift toward proinflammatory taxa by reducing mucosa-associated beneficial bacteria, which leads to increased permeability and barrier dysfunction [105]. Therefore, when studying the IBD microbiome, it is important to be mindful of NSAID usage, given its potential effects on gut mucosa and the intestinal microbiota [106].

Therapeutic agents used in IBD—such as corticosteroids, aminosalicylates, immunomodulators, biologics, and antibiotics—can themselves influence the gut microbiota, and vice versa. Medications may alter microbial diversity, metabolic pathways and products, and antibiotic resistance patterns, while microbial composition on the other hand can affect drug metabolism, efficacy, and toxicity. Understanding these bidirectional interactions is critical for optimizing treatment strategies and developing microbiota-targeted adjunctive therapies.

Thus, the study of medication–microbiota interactions in IBD patients offers a promising avenue to improve personalized medicine approaches, enhance therapeutic outcomes, and identify novel microbial or metabolite-based interventions. In the following section, we will investigate medication used in IBD patients and its effect on gut microbiota.

### 4.1. Corticosteroids

Microbiome changes: Oral or intravenous treatment with corticosteroids rapidly suppress systemic inflammation by broadly inhibiting pro-inflammatory cytokine production and immune cell activation. Beyond direct immunosuppression, corticosteroids alter the colonic microenvironment and can shift gut microbial communities in both animal models and in humans (including changes in diversity and relative abundances of key taxa in the intestinal tract [107]). These microbiota changes may influence mucosal barrier function and metabolite production (e.g., SCFAs), with potential downstream effects on intestinal mucosal healing and generalized susceptibility to infection. Thus, while steroids are highly effective for induction of disease remission in IBD patients, their effects on the microbiome might contribute to variability in response and to infection risk during therapy [107].

Blesl et al., 2024 [108] performed a prospective study of UC patients in active disease states who received systemic corticosteroids, which was associated with a longitudinal restoration of microbial composition and metabolic capacity. The study showed successful steroid treatment tended to shift the microbiome toward a healthier composition [108]. Guo et al., 2022 [109] conducted a study where two induction therapies were compared: exclusive enteral nutrition (EEN) [110] versus treatment with systemic corticosteroids. The microbiome analysis of serial CD patients’ fecal samples collected before and after induction therapy showed how treatment with EEN and corticosteroids, respectively, resulted in different trajectories of changes in the gut microbiome. Patients who received treatment with EEN tended to have larger shifts in community composition in their intestinal microbiome compared with patients treated with corticosteroids. This finding indicates that treatment with systemic steroids does change the intestinal microbiome in humans, but the magnitude and direction differ from that of dietary therapy [109]. Comparison of the composition of the microbiotas in the UC patients before and after induction therapy with glucocorticoid vs. fecal microbiota transplantation (FMT, colonoscopy) showed how both therapies significantly reduced the intestinal levels of the inflammation markers TNF-α and IL-10 [111], while treatment with steroids modify the ecosystem different compared with FMT. Li et al., 2023 [112] performed a rat-experiment study which showed that long-term prednisone treatment can cause fungal microbiota dysbiosis (disturbances in the composition of the fungal intestinal microbiome), which affects the ecological interaction between gut mycobiome and bacteriome in the rats. More studies are needed to investigate the effect of glucocorticoid on the gut microbiome in humans.

Non-microbiome side effects caused by corticosteroid therapy are described in Table 2 and include metabolic and endocrine [113], musculoskeletal [114], immunosuppression and risk of infections [115], neuropsychiatric [116], gastrointestinal disorders [117], dermatologic disorders [118], and adrenal disorders [119] side effects.

### 4.2. Aminosalicylates

Aminosalicylates such as mesalazine (5-ASA) are front-line therapies for mild-to-moderate UC cases and act primarily through topical anti-inflammatory effects in the colon. Animal studies show that treatment with 5-ASA can directly affect the gut bacteria, and the drug can be metabolized by the microbiota; conversely, microbiome composition appears to predict and can modulate the 5-ASA efficacy [120], Table 3. The microbial metabolism of 5-ASA may reduce its local bioavailability or alter the drug activity, and shifts in microbial community structure with 5-ASA treatment have been reported, Table 3. These bidirectional interactions suggest the gut microbiome both mediates and modifies the therapeutic effect of 5-ASA [121].

### 4.3. Immunomodulators

Thiopurines (azathioprine, 6-mercaptopurine) suppress the adaptive immunity, and the drugs are used for maintenance of disease remission in IBD patients. Recent research indicates that gut bacteria can influence the pharmacokinetics and efficacy of thiopurine [121]. For example, certain commensal bacteria decrease the conversion of the drug to active metabolites or otherwise reduce the drug bioavailability, which can potentially promote treatment failure [121]. Additionally, immunosuppression itself may alter microbiota composition indirectly via changes in the mucosal immunity. These interactions may partly explain interpatient variability in therapeutic response to various treatments, and it highlights the microbiome as a potential biomarker or interventional target to improve thiopurine treatment outcomes [121].

### 4.4. Biological Therapy

Biologic therapies (e.g., anti-TNF agents such as infliximab/adalimumab, anti-integrin vedolizumab) and anti-interleukin agents (Ustekinumab) [122] target specific inflammatory pathways in the intestinal tract and have transformed IBD management [122]. Multiple studies show that patients who respond to biological therapy often have distinct baseline intestinal microbiome features (higher α-diversity and greater abundance of butyrate-producing *Clostridiales* such as *Faecalibacterium prausnitzii*) compared with IBD patients classified as nonresponders, and it is reported how successful treatment with biological therapy can partially restore a “healthier” microbiome profile in the gut of IBD patients [123]. Conversely, the intestinal microbiome appears to influence biologic efficacy through the modulation [124] of mucosal immune activation, microbial metabolite production (short-chain fatty acid) [125], and intestinal mucosal healing capacity. Therefore, fecal microbial signatures are being investigated as predictive biomarkers for biologic response to various treatments and as targets for adjunctive microbiome therapies [126].

### 4.5. Janus Kinase (JAK) Inhibitors

JAK inhibitors [127] including tofacitinib (a pan-JAK inhibitor with predominant JAK1/3 activity) and upadacitinib (a selective JAK1 inhibitor) are increasingly used for moderate-to-severe ulcerative colitis (UC), particularly in biologic-exposed patients. Their impact on the gut microbiome has only recently been investigated, and although data remains limited, emerging findings indicate indirect microbiota-modulating effects mediated via inflammation control rather than direct antimicrobial action [127].

Early-phase multi-omics and sequencing studies suggest that clinical responders to JAK inhibition exhibit partial restoration of gut microbial diversity, especially increases in short-chain fatty acid (SCFA)-producing taxa such as *Faecalibacterium prausnitzii* and *Roseburia* spp., organisms typically depleted during active UC [127]. These improvements parallel reductions in inflammatory cytokines such as IFN-γ and IL-6, which may create a more permissive environment for beneficial anaerobes to recover [128].

A longitudinal profiling study of UC patients treated with tofacitinib found that responders demonstrated microbiome trajectories resembling remission associated states, including increased alpha diversity and reduced abundance of pro-inflammatory *Proteobacteria* [129]. Notably, non-responders did not show this pattern, suggesting that microbial shifts may reflect disease control rather than a direct drug effect. Overall, current evidence shows that JAK inhibitors do not directly alter microbial composition but rather normalize dysbiosis through rapid and profound suppression of intestinal inflammation, promoting the recovery of SCFA-producing commensals. However, data are still sparse, sample sizes are small, and more controlled prospective microbiome studies are needed.

### 4.6. Sphingosine-1-Phosphate (S1P) Receptor Modulator

S1P receptor modulators such as ozanimod represent a newer class of oral therapies for ulcerative colitis. These agents reduce gut-homing lymphocyte trafficking, thereby dampening mucosal inflammation [130]. Although direct human evidence on microbiome modulation is currently limited, emerging preclinical and translational studies suggest that S1P signaling can indirectly influence the gut microbial environment through immune–epithelial pathways [131].

Clinical trials of ozanimod in UC primarily evaluate clinical and endoscopic outcomes, but investigators have hypothesized that reduced inflammation may secondarily promote a more favorable microbial profile [132]. However, no published clinical study to date has systematically characterized microbiome shifts during S1P-modulator therapy, making this an important area for future research.

### 4.7. Early-Life Exposures and Their Influence on Gut Microbiota in IBD

A growing body of evidence shows that early-life exposures profoundly influence microbial development and may increase subsequent risk of IBD. Antibiotics given during infancy disrupt gut microbiome maturation, reduce diversity, and alter long-term colonization patterns [133]. Multiple cohort studies have linked childhood antibiotic exposure to increased IBD risk, particularly Crohn’s disease [134].

Perinatal factors:

The mode of delivery strongly shapes initial microbial colonization. Cesarean delivery is associated with delayed establishment of enriched anaerobic communities [135] and persistence of skin-associated taxa [134].

Breastfeeding promotes *Bifidobacterium*-dominant profiles that support immune regulation and epithelial tolerance [134]. These factors may influence long-term immune development relevant to IBD susceptibility [136].

Pediatric IBD:

Children diagnosed with IBD display more severe dysbiosis than adults, including reduced microbial diversity, lower abundance of butyrate-producing taxa, and increased Proteobacteria [136]. Because pediatric patients have fewer accumulated environmental confounders, these findings help identify early biological signatures associated with disease development.

### 4.8. Antibiotics

Antibiotics profoundly and often persistently disrupt the gut microbial diversity and community composition; broad-spectrum agents can deplete beneficial commensals (including butyrate producing taxa such as *Faecalibacterium prausnitzii* and *Roseburia* spp.) while promoting overgrowth of opportunistic or resistant organisms [137]. In patients suffering from IBD, antibiotics are used selectively (e.g., for infections, pouchitis, or perianal disease), but the use is associated with both short-term symptomatic changes such as diarrhea and vomiting and longer-term risks such as long-lasting dysbiosis in the intestinal microbiome.

In general, antibiotic exposure has been linked to an increased risk of new-onset IBD in epidemiologic studies, and antibiotics can worsen or complicate microbiome-mediated metabolic and immune functions in patients who have already been diagnosed with IBD [138]. At the same time, targeted antibiotic regimens can be therapeutic in certain IBD-related complications, underscoring that antibiotic–microbiome interactions are highly context-dependent. Epidemiological studies show that early-life and repeated antibiotic exposure significantly increase the risk of developing IBD, most likely by perturbing immune tolerance and intestinal barrier function [139]. Furthermore, antibiotics can exacerbate intestinal dysbiosis and impair the metabolic and immunologic regulatory pathways [140]. Conversely, targeted antibiotic regimens, such as metronidazole or ciprofloxacin in patients suffering from perianal localized CD, can be therapeutically beneficial in specific contexts [141]. These findings emphasize that antibiotic–microbiome interactions in IBD are highly context-dependent, capable of both therapeutic and detrimental side effects depending on timing, spectrum, and host factors.

## 5. Discussion

Diet and commonly prescribed medications are among the strongest and most influential factors shaping the composition and function of the gut microbiota. Multiple studies indicate that both diet and medication can act as key environmental triggers in the development of IBD in genetically predisposed individuals. Evidence supporting this includes findings that identical twins are often discordant for IBD, demonstrating that genetics alone cannot explain disease onset [142]. Furthermore, higher rates of IBD have been observed among second-generation immigrants, highlighting the strong impact of environmental exposures (particularly diet, lifestyle, and medication use) on disease risk [143]. An increased prevalence of IBD is seen among individuals living in developing countries, which indicates environmental factors such as food and medicine play a significant role in the disease triggers of IBD. It is important to note that eating and drinking, beyond their biological necessity, serve as important psychological and social roles: providing pleasure, comfort, and a sense of belonging [144]. Food is deeply intertwined with social and cultural identity and plays a central part in how people interact, celebrate, and connect with family, friends, and colleagues [25]. Patients suffering from IBD use different food-related strategies to control/manage intestinal symptoms, such as identifying trigger foods, following restrictive diets, control of portion size, and shortening of eating intervals, which might have consequences on the intake of nutrition. As a result of these behavioral strategies and limited knowledge on the effect of dieting on the disease, IBD patients’ food-related quality of life is also affected. Known beneficial diets rich in fiber, omega-3, prebiotics, and fermented foods will promote SCFA producers such as *Faecalibacterium prausnitzii* and *Roseburia*, which are associated with a healthy gut microbiome.

However, dietary tolerance varies greatly among patients with IBD, even foods generally considered beneficial. This variability makes it challenging to recommend a single dietary approach for all patients with IBD. Instead, personalized nutrition plans—developed in collaboration with dietitians or nutrition specialists—may yield more favorable outcomes, particularly in patients with CD. It is important to be cautious when providing dietary recommendations to IBD patients as a physician and ensure that patients do not replace or rely on commonly consumed processed foods or beverages, such as cola, which have been shown to exert pro-inflammatory effects on the gut.

Maintaining a balance in dietary intake is important, as excessive consumption of otherwise beneficial foods or prebiotics may lead to adverse and unwanted effects in IBD patients. For instance, overconsumption of inulin has been associated with liver dysfunction in experimental studies [84]. The studies link a Westernized diet to increased pathobionts such as *E. coli* and *Bilophila wadsworthia*, decreased levels of SCFA-producing bacteria, and a reduction in SCFA, which are linked to most of the autoimmune diseases, including IBD.

The intake of medication plays a key role in shaping our gut microbiome, specifically in early childhood, as evidence indicates that antibiotic usage, such as broad-spectrum β-lactams, during the first year of a newborn is linked to autoimmune diseases such as the development of IBD [145].

The medication in focus, when studying the IBD intestinal microbiome, is that used to promote and maintain disease remission in IBD patients, such as corticosteroids (e.g., prednisone, budesonide). The studies show that corticosteroids induce alteration in the gut microbiota, including metabolic disturbances, fungal microbiota dysbiosis, increased susceptibility to infection, and neuropsychiatric manifestations [112]. Treatment with corticosteroids significantly reduces TNF-α and IL-10 [111], which promotes disease remission in IBD patients. However, given the established importance of the gut microbiome in maintaining overall health, it is reasonable to question if the non-microbiome-related side effects of corticosteroid therapy—such as metabolic and endocrine [113] disturbances, immunosuppression, and increased infection risk [115] (Table 2)—may in fact be mediated, at least in part, induced by corticosteroid-related alterations in gut microbial composition and function. This potential microbiome-dependent mechanism warrants further investigation.

Anti-inflammatory Aminosalicylates (5-ASA) are metabolized by the gut bacteria, therefore only IBD patients with the right composition of the intestinal microbiome will experience 5-ASA efficacy [120,121]. The efficacy of immunomodulators, such as azathioprine, is linked to the composition of the gut bacteria, as certain commensal bacteria decrease conversion of the drug derivatives to active metabolites or otherwise reduce drug bioavailability, which is followed by treatment failure [121]. The studies indicate that immunosuppression itself caused by treatment with immunomodulators may alter microbiota composition indirectly via changes in mucosal immunity [121].

The research shows how the baseline composition of the intestinal microbiome is important, when treating IBD patients with biological therapy such as anti-TNF, anti-integrin, and anti-IL agents. The intestinal microbiome appears to influence the efficacy of biological therapy through the modulation of mucosal immune activation and microbial metabolite production in the gut [123,125]. Overall, the research reviewed reports how the majority of IBD drugs both shape and are shaped by the intestinal microbiome, which secondly affects drug bioavailability, efficacy, safety, and long-term mucosal outcomes in IBD patients. The baseline microbial composition is a predictor of the efficacy of biological therapy and anti-inflammatory drugs such as aminosalicylates. This enables a new therapeutic opportunity to enhance drug response or reduce toxicity by modulating the gut microbiome through diet and fecal microbiota transplantation. Future randomized controlled studies are warranted to assess the potential of fecal microbiota transplantation (FMT) to beneficially reshape the gut microbiome and thereby enhance the clinical efficacy and durability of biologic therapies in patients with IBD.

While our review initially discusses diet and pharmacotherapy separately, it is increasingly recognized that these factors interact in clinical practice, producing additive, synergistic, or competing effects on gut microbiota. For example, dietary interventions may modulate microbial composition in ways that enhance or counteract the effects of medications such as antibiotics, immunosuppressants, or biologics. These interactions can influence inflammation, metabolite production, and overall gut ecosystem resilience. Understanding the combined impact of diet and pharmacotherapy is crucial for designing personalized therapeutic strategies. Future studies should evaluate these interactions longitudinally in human cohorts to determine optimal combinations for restoring microbial balance and improving clinical outcomes in IBD.

## 6. Conclusions

Diet and medication are central modulators of gut microbiota in IBD, and their effects can be beneficial or detrimental depending on context, dosage, and timing. Understanding these mechanisms is essential for guiding precision nutrition and therapy. This review highlights the growing evidence that dietary interventions, pharmacotherapy, and fecal microbiota transplantation can modulate gut microbial composition and influence disease outcomes. However, significant knowledge gaps remain, particularly regarding integrative approaches that combine diet and medication, the limited clinical evidence for FMT in IBD, and the need for larger, well-controlled longitudinal studies. Future research should focus on personalized microbiota-targeted strategies, combined dietary and pharmacological interventions, and mechanistic studies in both human and animal models. The findings have practical implications for clinicians and dietitians, providing a scientific basis for optimizing dietary guidance, therapeutic decisions, and patient management in IBD.

## Figures and Tables

**Table 2:** Table shows corticosteroids associated extensive systemic side effects.

Category	Example Side Effects	Mechanism
Metabolic/Endocrine	Weight gain, Diabeetes, hypertension	Incresed Cluconeogenesis, insulin resistance [113]
Musculoskeletal	Osteoporosis, myopathy	Increased bone resorption, protein catabolism [114]
Infectious	TB, fungal, viral	Immunosuppression [115]
Neuropsychiatric	Mood swings, psychosis	Cortisol effects on CNS [116]
Gastrointestinal	Ulcers, hepatic steatosis	Mucosal thinning, metabolic [117]
Dermatologic	Bruising striae	Reduced collagen synthesis [118]
Adrenal	Adrenal insufficiency	HPA axis suppression [119]

**Table 3:** Mechanistic effect of after 5-ASA.

Mechanistic Target	Effect	Clinical Impact
NF-κB inhibition	Reduce pro-inflammatory cytokines	Reduces mucosal inflammation
PPAR-γ activiation	Increase Anti-inflammatory gene expression	Promotes mucosal healing
COX-2 inhibition	Reduce prostaglandins	Decreases edema and pain
ROS scavenging	Reduce Oxidative Stress	Protects epithelium
Barrier restoration	Incresed Tight-Junction proteins	Improves epithelial integrity
Microbiome modulation	Increased producers, decrease Proteobacteria	support remission

## Data Availability

No new data were created or analyzed in this study. Data sharing is not applicable to this article.

## Abbreviations

The following abbreviations are used in this manuscript:

IBD	Inflammatory bowel disease
CD	Crohn’s disease
UC	Ulcerative colitis
FMT	Fecal Microbiota Transplantation
PPI	proton pump inhibitor
